# Developmental Patterns in Autism and Other Neurodevelopmental Disorders in Preschool Children

**DOI:** 10.3390/children12020125

**Published:** 2025-01-24

**Authors:** Maria Eugenia Martelli, Federica Gigliotti, Federica Giovannone, Giuliana Lentini, Filippo Manti, Carla Sogos

**Affiliations:** Department of Human Neuroscience, Sapienza University of Rome, 00185 Rome, Italy; mariaeugenia.martelli@uniroma1.it (M.E.M.); federica.gig@uniroma1.it (F.G.); federica.giovannone@uniroma1.it (F.G.); giuliana.lentini@uniroma1.it (G.L.); filippo.manti@uniroma1.it (F.M.)

**Keywords:** developmental profile, adaptive profile, neurodevelopmental disorders, preschool children

## Abstract

Background: Neurodevelopmental disorders (NDDs) encompass an inclusive group of conditions that appear during the developmental period but continue to persist in adulthood, ranging from particular difficulties to a global impairment of social, cognitive, and emotional functioning. The developmental trajectories associated with these conditions are highly heterogeneous. This study aimed to analyze and compare developmental and adaptive profiles of preschool-aged children with different NDDs to better characterize their developmental trajectories. Methods: We analyzed data from the initial global evaluation of 196 children with NDDs (aged 20 to 71 months), enrolled in three subgroups: 108 with autism spectrum disorder (ASD), 52 with language disorder (LD), and 36 with mixed specific developmental disorder (MSDD). A comprehensive neuropsychiatric evaluation was performed using standardized tools (Griffiths-III, ADOS-2, VABS-II, and ADI-R), and the parents completed the DP-3 and the CBCL 1½-5. Results: Our results showed that all NDDs exhibited poor psychomotor skills, with children with ASD being the most impaired, although their profiles were comparable to those of MSDD in communication and motor areas. CBCL’s pervasive developmental problem scale has been shown to provide relevant information for distinguishing children with ASD. Furthermore, DP-3 and VABS-II measure highly differentiated developmental profiles of each diagnostic group. Conclusions: Our results highlighted the importance of including parents’/caregivers’ perspectives in defining children’s functioning and the possibility of using DP-3 as a screening tool for different neurodevelopmental disorders.

## 1. Introduction

Neurodevelopmental disorders (NDDs) encompass a wide range of conditions that affect brain development, leading to delays or impairments in cognitive, motor, linguistic, and socio-emotional domains [1,2]. These disorders, which can persist throughout life and significantly impact the quality of life, appear early in the child’s development, often before school age [3]. However, diagnosing these conditions in preschoolers poses significant challenges due to the complexity and high variability of early development, significant overlap across NDDs [4], and limitations in diagnostic tools that can lead to diagnostic uncertainty, delayed identification, or misdiagnosis.

Over the past 15 years, research has highlighted that developmental profiles in NDDs are highly heterogeneous, not following uniform patterns but instead representing dynamic continua shaped by a complex interplay of genetic, neurobiological, and environmental factors [5,6]. In preschool-aged children, identifying and understanding these trajectories is essential for early diagnosis and implementation of effective interventions tailored to the unique child’s needs, which can significantly influence developmental outcomes and quality of life [7].

Differential diagnosis between different NDDs in preschool-aged children is a complex and nuanced process that requires careful assessment of cognitive, linguistic, social, and behavioral domains to reveal strengths and weaknesses in different areas of development [4].

Autism spectrum disorder (ASD) is a disorder characterized by persistent deficits in social communication and interaction, as well as restricted and repetitive patterns of behavior, interests, or activities [3]. In this condition, there is significant variability in developmental trajectories, with early indicators often emerging in social communication and sensory–motor integration. Longitudinal studies suggest that some children exhibit developmental plateaus or regressions, particularly in language and social interaction domains [8].

Cognitive abilities in ASD are heterogeneous; a previous study found that up to 78% of preschool children with ASD had clear or suspected developmental delays and another 20% presented average or above-average cognitive levels, resembling the clinical profile of high-functioning autism [9]. On the other hand, some ASD children may display uneven skill development with strengths in specific areas (such as rote memory or visual–spatial processing) and challenges in executive functioning [10,11].

Verbal skills can vary widely, ranging from nonverbal communication to advanced vocabulary with atypical use. Language delays are quite common in ASD, with challenges in both expressive and receptive language, leading to diagnostic confusion with LD. However, children with ASD often exhibit pragmatic impairments and qualitative differences in language use, such as echolalia or unusual prosody, which are less typical in LD [12]. Nonverbal communication is also frequently affected.

Additionally, impairment in social reciprocity, joint attention (e.g., difficulties in sharing attention with others through pointing or eye contact), and emotional regulation, as well as repetitive behaviors (e.g., hand flapping or fixations on specific objects) and restricted interests, are hallmark features in preschool children with ASD [13], but they can be subtle or transient, particularly in children under the age of three, leading to delayed recognition of the disorder [8].

Language disorder (LD) involves significant and persistent difficulties with language acquisition and use, because of impairments in understanding or producing language, in the absence of intellectual disability, sensory impairments, or other medical conditions [3]. Children with LD had significant difficulties in areas highly dependent on language, such as communication and community participation; the severity of these weaknesses ranges from very mild to substantial enough to qualify as disabilities. On the other hand, these children exhibit strengths in areas less reliant on language, including domestic and personal life activities, play and leisure, coping in social situations, and gross motor skills [14].

While primary language disorder may not involve general cognitive delays, challenges in working memory and processing speed are often noted [15,16].

So, children with LD primarily struggle with the structure and use of language, while their social engagement and nonverbal communication skills are generally within the expected developmental range [17]. However, previous research has shown that language impairments can hinder social interactions, potentially resulting in social withdrawal or behavioral issues due to frustration or misunderstanding [12].

Mixed specific developmental disorder (MSDD), as defined by the International Statistical Classification of Diseases and Related Health Problems 10th Revision (ICD-10), describes a condition characterized by delays or difficulties across multiple developmental domains, including cognition, language, motor skills, social interaction, and behavioral regulation [18], without meeting the full criteria for a specific neurodevelopmental disorder. MSDD is not a distinct diagnostic category in current classifications, such as DSM-5-TR or ICD-11, but is often used clinically to describe heterogeneous developmental profiles in preschool-aged children when there is a significant impairment in the development of several functions without any single developmental domain being predominantly affected.

The main aim of this study was to identify and evaluate the developmental level and the adaptive profile of preschool-aged children with diagnoses of NDDs in order to better understand different developmental trajectories. Furthermore, we compared the profiles of different clinical groups in order to verify any differences between these subpopulations that could have implications for diagnostic and therapeutic strategies.

## 2. Materials and Methods

### 2.1. Participants

This study involved 196 preschoolers (M:F = 163:33), ranging from 20 and 71 months (40.7 ± 10.2), with neurodevelopmental disorders.

Participants were recruited among children referred to the Outpatient Service for Neurodevelopmental Disorders of the Department of Human Neuroscience at Sapienza University of Rome, due to difficulties in interaction, communication, and behavior, between 2021 and 2024.

Inclusion criteria were (a) having an age between 1 and 72 months, (b) having received a clinical diagnosis of NDD, encoded according to the ICD-10 classification (currently adopted in the Italian health care system), (c) being in the care of their parents, and (d) not taking drug treatment or practicing other types of therapy.

Children who had a diagnosis of intellectual disability or associated medical conditions (e.g., neurocutaneous or other genetic syndromes, epilepsy, traumatic brain injury, significant sensory or motor deficits, other chronic diseases, or reactive attachment disorders) were excluded. Further exclusion criteria were the inability to perform a structured evaluation of development, due to severe behavioral problems, and the lack of sufficient knowledge of Italian by parents to complete the entire assessment.

### 2.2. Procedure

The participants underwent a comprehensive diagnostic evaluation, including medical history, developmental level assessment, and observational behavior analysis, during which the disorder was confirmed. Both parents completed a symptom checklist (Child Behavior Checklist for Ages 1½-5 years—CBCL 1½-5) to jointly assess their child’s emotional and behavioral problems, the Vineland Adaptive Behavior Scale, 2nd Edition (VABS-II), to evaluate the child’s adaptive functioning, and the Developmental Profile-3 (DP-3) to investigate the child’s developmental profile. Specific instruments (Autism Diagnostic Observation Schedule, 2nd Edition—ADOS-2, and Autism Diagnostic Interview-Revised—ADI-R) were used to assess autistic symptoms when clinically indicated.

According to the ICD-10 classification, the total sample was divided into 3 clinical groups: children with LD, MSDD, or ASD.

#### 2.2.1. Developmental Profile

All participants were assessed using the Italian adaptation [19] of the Griffiths Scales of Child Development, 3rd Edition (Griffiths-III) [20], which was developed as an evaluation instrument for children aged up to 72 months of age. The administration takes approximately an hour and a half, depending on the clinician’s expertise and the child’s physical and emotional disposition.

Griffiths-III provides a detailed profile of child psychomotor development (general developmental quotient—GDQ) and allows the assessment of five domains of development: foundations of learning (subscale A), language and communication (subscale B), eye and hand coordination (subscale C), personal–social–emotional (subscale D), and gross motor (subscale E). Each item is scored as a pass (+1) or a fail (0). Raw scores, obtained from the sum of the items passed in each scale, are calculated to determine the Age Equivalent, Scaled Score, and development quotient (DQ), according to the sex and age of patients. In this study, we considered only DQs.

The DP-3 [21] is a standardized instrument designed to evaluate the development and functioning of children aged up to 12 years old, providing information about strengths and weaknesses. It is the updated and revised version of Developmental Profile-II and consists of either an interview or a parent/caregiver checklist; for this study, we used the second version.

It has 180 items, each describing a particular skill, with yes/no questions, assessing 5 key areas of development: physical (35 items), adaptive behavior (37 items), social–emotional (36 items), cognitive (38 items), and communication (34 items). Each scale provides a raw score which is converted into a standard score; the sum of the standard scores of the five domains is finally converted into the general development score, according to the norm tables. Italian adaptation of the DP-3 showed good reliability, both in terms of internal consistency (with split-half coefficients ranging from 0.64 to 0.96) and test–retest reliability (correlation range: 0.866–0.968) [22].

#### 2.2.2. Adaptive Function Profile

To characterize the adaptive functioning of the participants, the survey interview form of the VABS-II [23] was administered to parents. This semi-structured interview is specifically designed to assess the adaptive level of functioning as a standardized measure in individuals aged up to 90 years old.

The VABS-II measures 11 subdomains grouped into four broad domain composites (communication, daily living skills, socialization, and motor skills). Each item can be scored as 0 (never), 1 (sometimes), or 2 (usually or often). The broad domain raw scores, calculated by summing the raw scores from each subdomain, are converted into standard scores which are then combined to provide an adaptive behavior composite (ABC) score, expressing the individual’s global adaptive functioning. The Italian adaptation of the scale showed good/excellent internal consistency reliability for broad domains and ABC (split-half coefficient range: 0.80–0.97) [24].

#### 2.2.3. Emotional and Behavioral Profile

The CBCL 1½-5 [25] is a parent-report screening tool that assesses behavioral, emotional, and social functioning in preschoolers. It consists of 100 items rated on a 3-point scale: 0 = not true, 1 = somewhat true or sometimes true, or 2 = very true or often true. Item raw scores are used to compute T-scores for seven syndromic scales (emotionally reactive, anxious/depressed, somatic complaints, withdrawal, sleep problems, attention problems, and aggressive behavior), five DSM-oriented scales (affective problems, anxiety problems, pervasive developmental problems, attention deficit/hyperactivity problems, and oppositional defiant problems) and three summary scales (internalizing, externalizing, and total). In the present study, we used the Italian version of the scale and considered the T-scores of the DSM-oriented scales and the summary scales.

The CBCL 1½-5 has shown good psychometric properties, including high internal consistency (approximately 75% of scales having a Cronbach’s alpha of 0.78 or greater) and strong test–retest reliability (*r* value range: 0.74–0.90).

#### 2.2.4. Signs and Symptoms of ASD

Specific standardized instruments were administered in the differential diagnosis of patients with autistic features.

The ADOS-2 [26] is a standardized assessment instrument designed to evaluate individuals with a suspected diagnosis of ASD. It consists of a series of semi-structured activities and prompts that allow researchers to observe behaviors related to communication, social interaction, play, and restricted or repetitive behaviors.

This clinical instrument is widely regarded as the “gold standard” for ASD assessment and it is divided into five different 40–60 min modules, each tailored to different age groups and language capabilities (Toddler Module, for children aged 12 to 30 months who have limited or no use of phrase speech; Module 1, for children aged 31 months or older who have limited or no use of phrase speech; Module 2, for children who use phrases but not fluently; Module 3, for children and young adolescents with fluent speech; and Module 4, for older adolescents and adults who are verbally fluent).

The ADOS-2 yields calibrated severity scores (CSSs) for the two domains of social affect and restricted and repetitive behaviors, as well as for the total score.

In this study, either the Toddler Module, Module 1, or Module 2 were used. Furthermore, the severity of the autistic symptoms was measured with the total CSS of the ADOS-2, ranging from 1 to 10. The Toddler Module CSS reported in the current study was computed based on Esler et al. (2015) [27] to allow direct comparison with other modules of the ADOS-2.

The ADI-R [28] is a 93-item standardized structured interview, which explores core symptomatic areas of autism: language development, social interaction, and communication abilities as well as play, interests, and behaviors. The items are split into 4 scales: (A) reciprocal social interaction, (B) communication and language, (C) restricted, repetitive, and stereotyped behaviors, and (D) abnormality of development evident at or before 36 months.

In the present study, we used the Italian version [29], and we took into account the scores of A, B, and C scales.

### 2.3. Data Analysis

All statistical analyses were performed using version 25.0 of the IBM Statistical Package for Social Science software (SPSS, Inc., Chicago, IL, USA, IBM, Somers, NY, USA).

Descriptive statistics were applied to the sociodemographic and clinical data. Continuous variables were presented as mean ± standard deviation (SD). Normality was assessed with the Kolmogorov–Smirnov test. Correlation analyses were performed using Spearman’s correlation coefficient. For continuous data, an independent sample *t*-test and a one-way analysis of variance (ANOVA) with post hoc comparison (Bonferroni) were used.

The dimensional effect of clinical variables on outcome was evaluated by linear regression analysis (step-wise method). A *p*-value <0.05 represented statistical significance for all tests.

## 3. Results

### 3.1. Sociodemographic Data and Clinical Features

The research sample consisted of 196 children, of which 52 (26.5%) met the specified criteria for a diagnosis of LD, 36 (18.4%) for MSDD, and 108 (55.1%) for ASD. The mean age and gender ratio did not differ between the three clinical groups (F = 1.186, *p* = 0.308; F = 0.633, *p* = 0.589).

Developmental level, estimated by Griffiths-III GDQ, was significantly different across the groups (F = 69.146; *p* < 0.01); the post hoc analysis showed that GDQ was significantly higher in the LD group compared to the MSDD group (*p* < 0.01) and the ASD group (*p* < 0.01), while there was no significant difference between the MSDD and the ASD group (*p* = 0.061). A significant difference among groups was also found in all Griffiths-III subscales: foundations of learning (F = 39.087; *p* < 0.01), language and communication (F = 42.148; *p* < 0.01), eye and hand coordination (F = 50.154; *p* < 0.01), personal–social–emotional (F = 57.898; *p* < 0.01), and gross motor (F = 17.050; *p* < 0.01). Significantly higher mean scores were found in the LD group than in the MSDD or the ASD group while there were no statistically significant differences between ASD and MSDD (see Table 1).

In terms of parent ratings of children’s developmental functioning, assessed by the DP-3 checklist, we found significant differences among the three groups in the DP-3 general development quotient (F = 30.111; *p* < 0.01); a significant difference among groups was also found in all mean DP-3 scores (physical: F = 5.387, *p* < 0.01; adaptive behavior: F = 16.117, *p* < 0.01; social–emotional: F = 22.378, *p* < 0.01; cognitive: F = 30.978, *p* < 0.01; and communication: F = 23.606, *p* < 0.01). Post hoc analysis highlighted significantly higher mean scores in the LD group than in the MSDD or ASD group in the cognitive and communication scales. On the physical scale, the LD group obtained significantly higher scores than the ASD group (*p* < 0.01); no statistically significant differences were highlighted between the LD and the MSDD group and between the MSSD and the ASD group. On the adaptive behavior scale, significantly higher scores were found in the LD group compared to ASD (*p* < 0.01); a significant difference was also found between the MSDD and ASD group (*p* = 0.023). On the socio-emotional scale, higher mean scores were highlighted in the LD group than in the MSDD (*p* < 0.01) or the ASD group (*p* < 0.01); no statistically significant differences were found between the MSDD and the ASD group. Regarding the general development quotient, the LD group obtained significantly higher scores than the MSDD (*p* < 0.01) and the ASD group (*p* < 0.01); a statistically significant difference was also highlighted between the MSDD and the ASD group (*p* = 0.010) (see Table 2).

Analysis of mean VABS-II scores showed significant differences among the three groups in all domains (communication: F = 29.854, *p* < 0.01; daily living skills: F = 45.736, *p* < 0.01; socialization: F = 43.111, *p* < 0.01; and motor skills: F = 8.869, *p* < 0.01) and in the adaptive behavior composite score (ABC) (F = 44.636; *p* < 0.01). Subsequent post hoc analyses revealed that mean communication, daily living skills, socialization, and ABC scores were higher in the LD group compared to the MSDD group (all *p* < 0.01) or the ASD group (all *p* < 0.01); there was also a significant difference between the MSDD and the ASD group. In the motor skills domain, significantly higher scores were found in the LD group compared to the MSDD (*p* = 0.030) and the ASD group (*p* < 0.01) while there were no statistically significant differences between the MSDD and the ASD group (see Table 3).

The Supplementary Materials Figure S1 provides a comparison of developmental patterns among the three clinical groups.

As can be seen in Table 4, on the CBCL 1½-5, a significant difference among groups was found in mean T-scores for all composite scales (internalizing: F = 10.186, *p* < 0.01; externalizing: F = 3.197, *p* = 0.043; and total: F = 5.279, *p* < 0.01) and for three DSM-oriented subscales (affective problems: F = 4.804, *p* < 0.01; pervasive developmental problems: F = 15.539, *p* < 0.01; and attention deficit/hyperactivity problems: F = 3.109, *p* = 0.047). The post hoc analysis highlighted significant differences between the LD group compared to the ASD group. Statistically significant differences were found between the MSDD and the ASD group only in the pervasive developmental problems subscale (*p* = 0.011).

In terms of autistic features, as expected, the mean ADOS-2 CSS in the group with ASD was significantly higher than in the group with MSDD (*p* < 0.01). Similarly, on the ADI-R, all scores were significantly higher in children with ASD (all *p* < 0.01) (see Table 5).

### 3.2. Correlation Analyses

Bivariate correlational analysis evidenced strong relationships between assessment tools. The Griffiths-III GDQ was negatively correlated with measures of autistic symptoms, such as ADOS-CSS (r = −0.53, *p* <0.01) and ADI-R (reciprocal social interaction: r = 0.54, *p* < 0.01; communication and language: r = 0.54, *p* < 0.01; and restricted, repetitive, and stereotyped behavior: r = 0.32, *p* < 0.01), and with the CBCL 1½-5 total score (r = −0.19, *p* < 0.01), However, the GDQ was positively correlated with the VABS-II ABC (r = −0.65, *p* < 0.01) and the DP-3 general development score (r = −0.66, *p* < 0.01). Adaptive functioning, measured using VABS-II, showed strong correlations with all assessment tools. Only the motor skills domain did not significantly correlate with the ADOS-2 CSS (r = −0.10, *p* = 0.2). Conversely, CBCL 1½-5 correlational results were less consistent among the scales. Considering the CBCL 1½-5 total score, no significant correlation was observed with the ADOS-2 CSS (r = −0.12, *p* = 0.13), but it was positively correlated with the ADI-R domains (reciprocal social interaction: r = 0.42, *p* < 0.01; communication and language: r = 0.39, *p* < 0.01; and restricted, repetitive, and stereotyped behavior: r = 0.38, *p* < 0.01) and negatively correlated with the Griffiths-III GDQ (r = −0.19, *p* < 0.01), VABS-II ABC (r = −0.44, *p* < 0.01), and the DP-3 general development scores (r = −0.4, *p* < 0.01). Moreover, as expected, we found strong positive relationships between all DP-3 subdomains and measures of psychomotor and adaptive functioning, as well as negative correlations with the ADOS-2 CSS and the ADI-R scales. Only the DP-3 physical domain did not correlate with ADOS-2 CSS (r = −0.09, *p* = 0.24).

### 3.3. Predictor of Adaptive Functioning and General Developmental Quotient

A first step-wise method was applied with VABS-II ABC scores as the dependent variable and Griffiths-III GDQ, DP-3 general development scores, CBCL 1½-5 externalizing, CBCL 1½-5 internalizing, and CBCL 1½-5 total scores as independent variables across clinical groups. The model was significant, explaining, respectively, 55% of the variance in VABS-II ABC scores in LD children (R^2^ = 0.55, *p* < 0.01), 54% in the MSDD sample (R^2^ = 0.54, *p* < 0.01), and 36% in the ASD group (R^2^ = 0.36, *p* < 0.01).

A second step-wise method was applied with Griffiths-III GDQ as the dependent variable and DP-3 general development scores, ADOS-2 CSS, CBCL 1½-5 externalizing, CBCL 1½-5 internalizing, and CBCL 1½-5 total scores as independent variables. We found a significant effect of the DP-3 general development scores, as well as ADOS-2 CSS on Griffiths-III GDQ in ASD children (R^2^ = 0.50, *p* = 0.02).

## 4. Discussion

Neurodevelopmental disorders (NDDs) are highly heterogeneous; however, they share significant overlaps in both primary and secondary symptoms. Especially in early childhood, children exhibit limited verbal abilities, and this may mask distinctive features. This overlap often makes NDDs appear similar, and diagnosing might be more challenging; for example, many early autistic behaviors overlap with those linked to language delays or intellectual disability [4]. This underscores the importance of selecting appropriate clinical instruments or combinations of instruments to identify differences between NDDs and accurately capture their developmental profiles.

In this study, we administered both screening and diagnostic tools to describe the developmental profiles of ASD, LD, and MSDD in children aged from 0 to 72 months, identifying both differences and similarities. The assessment tools were varied in their levels of structuring, ranging from structured performance tests to parent questionnaires, and consisted of direct and reported measures about children’s psychomotor development and well-being.

The initial analysis revealed that, on average, LD children’s general developmental quotient fell into a lower average range, as was described in the literature [30]. However, they achieved higher scores across all domains of Griffiths-III and in all assessment instruments administered, when compared to other diagnostic groups.

They also exhibited fewer adaptive and socio-emotional problems according to VABS-II and CBCL 1½-5 total scores. These findings partially diverged from the existing literature, which suggests that children with language disorders are at risk for psychosocial difficulties or morbidity [31,32,33,34,35]. We could explain this discrepancy by the fact that many of these works investigated socio-emotional problems in adolescence or late childhood, focusing on the effects of persisting language problems during school age or co-occurrence of low IQ. Conversely, the mean age of our sample was only 40.67 months and there was no significant evidence of strong correlations between language delays and behavioral problems in toddlers, except for withdrawn scores [36]. Indeed, caregivers frequently remain unaware of children’s socio-emotional or behavioral difficulties until 36 months of age, when children attend school and parents receive feedback from teachers.

However, considering information from the DP-3, it emerged that LD children had delayed or below-average developmental profiles, with clinical difficulties not only in communication but also in adaptive, socio-emotional, and cognitive functioning.

Therefore, we might assume that DP-3 provided a more detailed and reliable picture of LD children’s skills. Indeed, DP-3 proved to also be an effective clinical tool for assessing expressive speech development disorders in preschool children [37].

Children with ASD exhibited the most impaired profile, showing the lowest scores in all domains of Griffiths-III, VABS-II, and DP-3, as well as the highest scores in CBCL 1½-5 domains. However, no significant differences were found in Griffiths-III quotients compared to the MSDD group in communication and motor fields.

The literature reported a prevalence of approximately 50% of global developmental delay or intellectual disability in preschoolers with ASD [38,39], alongside impairments in language, cognitive, and motor fields compared to neurotypical peers [40,41,42,43]. Autistic children frequently demonstrate difficulties concerning phonological and visuospatial working memory, executive functions, and Theory of Mind [40,44,45,46,47], which significantly impact multiple domains of development and adaptive functioning, as it was confirmed by Griffiths-III, VABS-II, and DP-3 scores. According to VABS-II and DP-3, individuals with ASD showed difficulties mostly in communication and a highly delayed developmental profile. Moreover, the relationship between IQ, language abilities, and adaptive functioning was well documented [48,49]. VABS-II confirmed the results of previous studies [50], revealing the following pattern: motor skills scores > daily living skills scores > socialization scores > communication scores. All domain standard scores positively correlated with Griffiths-III scale scores and negatively with ADOS-2 CSS. The only exception was the Vineland motor domain, which did not correlate with ADOS-2 CSS. When analyzing the adaptive profiles of other clinical groups, we did not observe the same pattern. LD children stood out for their higher daily living skills scores, followed by motor skills scores, social skills scores, and communication scores, though none of these scores fell into the clinical range. Conversely, MSDD children achieved good motor skills, comparable to those of ASD children, followed by daily living skills, social skills, and communication skills. All adaptive profiles significantly differed among diagnoses, except in the motor domain, where no differences were found between ASD and MSDD children. Similarly, the DP-3 physical domain did not significantly differentiate between these groups and no correlations were observed with ADOS-2. The motor skills domain was comparable among ASD and MSDD children, and we hypothesized that our assessment tools might lack the sensitivity needed to detect subtle differences between these profiles, or that motor skills did not characterize their developmental profiles in early childhood.

In ASD impairments, cognitive and social functioning are often combined with disruptive behaviors, such as conduct problems, physical and verbal aggression, and self-injuring [51]. Disruptive behaviors are very common and negatively impact the quality of life [52,53,54], reducing cooperative attitude, escaping demands, and gaining access to preferred objects or to restricted and repetitive interests [55,56]. We thought that these common conduct problems could interfere with completing tests of Griffiths-III and contribute to lowering scores and adaptive functioning [44].

Regarding the MSDD, we found that their Griffiths-III scores differed significantly from those of LD children and ASD children, except for communication and motor skills.

Interpreting these differences is challenging because of the limited and available literature.

Few studies have explored MSDD functioning and developmental profiles, though interest in their psychiatric comorbidities is increasing [57].

However, we hypothesized that the similarity in developmental profiles might be explained by the frequent application of the MSDD diagnosis to children previously identified as being at risk for autism spectrum disorders. ICD-10, indeed, describes the mixed specific disorder as “*a residual category for disorders in which there is some admixture of specific developmental disorders of speech and language, of academic skills, and motor function, but in which none predominates sufficiently to constitute the principal diagnosis*” [18].

Similar to ASD, mixed specific disorders involve impairments in more than one developmental area, threatening the typical developmental trajectory. However, in these cases, there is not a selective impairment of communication skills or other autistic behavioral anomalies, such as restricted and repetitive interests.

ADOS-2 and ADI-R are rarely administered in case of suspected language disorders but are used when ASD and mixed specific disorders are suspected. These tools are considered highly sensitive in detecting autistic symptoms and our results underscore their crucial role in distinguishing children with ASD and MSDD. As previously reported [44], our study confirmed that ADI-R and ADOS-2 CSS were significantly higher in children with ASD compared to those with mixed developmental disorders. Furthermore, these scores were negatively correlated with Griffiths-III, VABS-II, and DP-3 scores.

From a cross-diagnostic perspective, we found that the Griffiths-III scores efficiently differentiated children with language disorders from those with mixed developmental and ASD. However, it did not fully capture differences between MSDD and ASD profiles across all domains, such as communication and motor skills. Nevertheless, Griffiths-III showed significant correlations with adaptive functioning, developmental profiles, ADOS-2, and ADI-R scales (except for the restricted, repetitive, and stereotyped behavior scale of ADI-R). These findings are consistent with previous studies, further validating its usefulness as an essential tool for neurodevelopmental assessment.

Regarding CBCL 1½-5 scores, we observed consistency across the diagnostic groups, and only the pervasive developmental problem (DSM-PDP) scale detected differences between diagnoses.

This result confirmed findings in the literature, which provided evidence of the utility of CBCL1½-5 in identifying children with ASD in Level 1 screening. In particular, the DSM-PDP scale effectively distinguished ASD children from neurotypicals, but also from children with other psychiatric disorders [58,59,60,61]. We observed significant differences in the DSM-PDP score between ASD children and other NDDs, but not between MSDD and LD children. We hypothesized that the DSM-PDP is more sensitive to ASD compared to other NDDs and that the lack of significant differences between LD and MSDD children could be attributed to the limited sample size.

Then, we found that VABS-II and DP-3 scores were significantly correlated with the scores of each administered instrument. This suggested that adaptive functioning and developmental profiles were closely tied to developmental quotients, socio-emotional well-being, and autistic symptoms. These clinical assessment instruments proved highly sensitive in differentiating the diagnostic group, creating different profiles for each one.

Notably, regression analysis revealed that a significant portion of the variance in adaptive function across neurodevelopmental disorders and, in general, developmental scores of ASD children could be explained by DP-3. We assumed that DP-3 could be an efficient measure of development, bridging the gap between performance tests and clinical settings by providing information about the everyday functioning of patients.

## 5. Conclusions

The present study provided a descriptive analysis of the most common developmental disorders in early childhood, combining different types of clinical assessment tools: performance-based structured tests, semi-structured observation, semi-structured interviews, and questionnaires. We integrated information from clinical and natural settings to depict reliable, comprehensive, and detailed developmental profiles, highlighting strengths, weaknesses, and specific characterization aspects. We investigated multiple domains of global functioning, including adaptive, psychomotor, and socio-emotional functioning, and we identified overlaps between diagnostic groups to evaluate which clinical instruments were most efficient in distinguishing clinical patterns.

According to our results, all NDDs exhibited low psychomotor skills, with ASD children being the most compromised, although their profiles were comparable with those of MSDD in communication and motor areas. These findings were consistent with other clinical instrument results, which correlated with Griffiths-III scores, except for CBCL 1½-5 measures, likely due to the early age of the children and the sample size.

Only CBCL’s pervasive developmental problem scale provided relevant information in distinguishing ASD children. However, DP-3 and VABS-II measures highly differentiated developmental profiles of each diagnostic group and they were significantly correlated with performance-based tests. These results highlighted the importance of including parents’/caregivers’ perspectives in defining children’s functioning and the possibility of using DP-3 as a screening tool for different neurodevelopmental disorders. DP-3 could be a very useful assessment tool: its intuitive dichotomous structure allows for the investigation of all development domains, requiring minimal resources.

## 6. Limitations

Although this study offered a detailed descriptive analysis and valuable insights, certain limitations should be acknowledged. First, while the sample size was sufficient, the diagnostic groups were unbalanced. ASD children were more numerous than those in other groups and females were underrepresented. Future studies could include an equal number of children across all clinical groups and reduce the predominance of males. Second, we did not observe concordance regarding motor differences between children with ASD and MSDD. Although specific assessment instruments are available, their sensitivity and specificity may be reduced in preschool children with NDDs due to peculiar difficulties in behavior, comprehension, and imitation. Moreover, the limited literature on MSDD hindered deep analysis of developmental profiles and restricted comparison with other diagnostic categories. So, it was challenging to draw strong inferences about MSDD children’s functioning. Finally, we did not differentiate children based on their cognitive functioning. However, future longitudinal follow-up studies should discern high- and low- functioning children and evaluate the progression of their development profiles.

## Figures and Tables

**Table 1:** Between-group comparisons of the Griffiths Scales of Child Development, 3rd Edition.

Griffiths-III Quotients, Mean (SD)	Total Sample (n = 196)	LD Group (n = 52)	MSDD Group (n = 36)	ASD Group (n = 108)	Post Hoc	*p*-Value
FL	65.75 (28.47)	90.36 (15.01)	64.39 (21.96)	54.36 (28.05)	LD vs. ASD	<0.01 *
					LD vs. MSDD	<0.01 *
					MSDD vs. ASD	0.097
LC	50.89 (27.26)	75.46 (18.97)	47.22 (24.59)	40.28 (23.94)	LD vs. ASD	<0.01 *
					LD vs. MSDD	<0.01 *
					MSDD vs. ASD	0.348
EHC	66.95 (30.74)	96 (21.83)	64.75 (22.99)	53.69 (27.08)	LD vs. ASD	<0.01 *
					LD vs. MSDD	<0.01 *
					MSDD vs. ASD	0.069
PSE	56.98 (27.47)	84.33 (19.89)	53.97 (21.27)	44.81 (22.88)	LD vs. ASD	<0.01 *
					LD vs. MSDD	<0.01 *
					MSDD vs. ASD	0.092
GM	78.67 (22.08)	92.86 (15.32)	73.55 (22.17)	73.53 (21.97)	LD vs. ASD	<0.01 *
					LD vs. MSDD	<0.01 *
					MSDD vs. ASD	1.000
GDQ	54.67 (26.83)	82.86 (16.01)	51.44 (18.94)	42.17 (22.90)	LD vs. ASD	<0.01 *
					LD vs. MSDD	<0.01 *
					MSDD vs. ASD	0.061

LD = language disorder; MSDD = mixed specific developmental disorder; ASD = autism spectrum disorder; SD = standard deviation; Griffiths-III = Griffiths Scales of Child Development, 3rd Edition; FL = foundations of learning; LC = language and communication; EHC = eye and hand coordination; PSE = personal–social–emotional; GM = gross motor; GDQ = general development quotient. * *p* < 0.05.

**Table 2:** Between-group comparisons of the Developmental Profile-3.

DP-3 Scores, Mean (SD)	Total Sample (n = 196)	LD Group (n = 52)	MSDD Group (n = 36)	ASD Group (n = 108)	Post Hoc	*p*-Value
Physical	79.65 (20.37)	87.35 (17.61)	78.25 (22.15)	76.42 (20.20)	LD vs. ASD	<0.01 *
					LD vs. MSDD	0.110
					MSDD vs. ASD	1.000
Adaptive behavior	74.66 (20.94)	86.23 (16.15)	78.08 (20.37)	67.95 (20.61)	LD vs. ASD	<0.01 *
					LD vs. MSDD	0.166
					MSDD vs. ASD	0.023 *
Social–emotional	62.52 (25.30)	79.79 (23.93)	63.36 (23.53)	53.93 (22.47)	LD vs. ASD	<0.01 *
					LD vs. MSDD	<0.01 *
					MSDD vs. ASD	0.101
Cognitive	60.77 (28.10)	82.19 (21.11)	63.19 (29.39)	49.66 (24.37)	LD vs. ASD	<0.01 *
					LD vs. MSDD	<0.01 *
					MSDD vs. ASD	0.014 *
Communication	53.58 (26.21)	70.98 (22.80)	57.42 (27.36)	43.92 (22.66)	LD vs. ASD	<0.01 *
					LD vs. MSDD	0.026 *
					MSDD vs. ASD	0.010 *
General development	54.90 (25.69)	74.11 (21.63)	57.61 (26.62)	44.74 (21.49)	LD vs. ASD	<0.01 *
					LD vs. MSDD	<0.01 *
					MSDD vs. ASD	0.010 *

LD = language disorder; MSDD = mixed specific developmental disorder; ASD = autism spectrum disorder; SD = standard deviation; DP-3 = Developmental Profile-3. * *p* < 0.05.

**Table 3:** Between-group comparisons of the Vineland Adaptive Behavior Scale, 2nd Edition.

VABS-II Scores, Mean (SD)	Total Sample (n = 196)	LD Group (n = 52)	MSDD Group (n = 36)	ASD Group (n = 108)	Post Hoc	*p*-Value
Communication	73.86 (15.81)	85.83 (11.16)	74.89 (14.33)	67.75 (14.88)	LD vs. ASD	<0.01 *
					LD vs. MSDD	<0.01 *
					MSDD vs. ASD	0.025 *
Daily living skills	81.46 (14.39)	94.23 (11.94)	82.33 (11.78)	75.02 (11.95)	LD vs. ASD	<0.01 *
					LD vs. MSDD	<0.01 *
					MSDD vs. ASD	<0.01 *
Socialization	77.98 (12.99)	89.30 (10.54)	78.67 (9.53)	72.30 (11.40)	LD vs. ASD	<0.01 *
					LD vs. MSDD	<0.01 *
					MSDD vs. ASD	<0.01 *
Motor skills	82.89 (14.30)	89.67 (14.23)	81.91 (13.88)	79.95 (13.48)	LD vs. ASD	<0.01 *
					LD vs. MSDD	0.030 *
					MSDD vs. ASD	1.000
ABC	76.38 (12.87)	87.94 (9.82)	79.19 (10.32)	70.88 (11.21)	LD vs. ASD	<0.01 *
					LD vs. MSDD	<0.01 *
					MSDD vs. ASD	0.032 *

LD = language disorder; MSDD = mixed specific developmental disorder; ASD = autism spectrum disorder; SD = standard deviation; VABS-II = Vineland Adaptive Behavior Scale, 2nd Edition; ABC = adaptive behavior composite. * *p* < 0.05.

**Table 4:** Between-group comparisons of the Child Behavior Checklist for Ages 1½-5 years.

CBCL 1½-5 T-Scores, Mean (SD)	Total Sample (n = 196)	LD Group (n = 52)	MSDD Group (n = 36)	ASD Group (n = 108)	Post Hoc	*p*-Value
DSM-oriented scales						
Affective problems	56.92 (7.38)	54.75 (6.41)	55.83 (5.51)	58.33 (8.06)	LD vs. ASD	0.011 *
					LD vs. MSDD	1.000
					MSDD vs. ASD	0.223
Anxiety problems	56.83 (8.56)	55.33 (7.73)	56.64 (8.71)	57.62 (8.86)	LD vs. ASD	0.341
					LD vs. MSDD	1.000
					MSDD vs. ASD	1.000
Pervasive developmental problems	66.54 (10.14)	61.21 (9.21)	64.47 (9.37)	69.80 (9.61)	LD vs. ASD	<0.01 *
					LD vs. MSDD	0.341
					MSDD vs. ASD	0.011 *
Attention deficit/hyperactivity problems	57.61 (7.06)	55.65 (5.95)	57.50 (7.59)	58.59 (7.23)	LD vs. ASD	0.041 *
					LD vs. MSDD	0.674
					MSDD vs. ASD	1.000
Oppositional defiant problems	55.28 (6.80)	53.96 (7.80)	54.28 (6.04)	56.25 (6.94)	LD vs. ASD	0.138
					LD vs. MSDD	1.000
					MSDD vs. ASD	0.391
Composite scales						
Internalizing	57.63 (10.55)	52.85 (11.12)	56.28 (8.94)	60.38 (9.92)	LD vs. ASD	<0.01 *
					LD vs. MSDD	0.355
					MSDD vs. ASD	0.108
Externalizing	55.80 (10.21)	53.13 (10.31)	55 (9.43)	57.35 (10.21)	LD vs. ASD	0.043 *
					LD vs. MSDD	1.000
					MSDD vs. ASD	0.684
Total	57.51 (10.82)	53.92 (10.65)	56.42 (9.18)	59.60 (10.99)	LD vs. ASD	<0.01 *
					LD vs. MSDD	0.837
					MSDD vs. ASD	0.359

LD = language disorder; MSDD = mixed specific developmental disorder; ASD = autism spectrum disorder; SD = standard deviation; CBCL 1½-5 = Child Behavior Checklist for Ages 1½-5. * *p* < 0.05.

**Table 5:** T-test comparison between MSDD and ASD group (Autism Diagnostic Observation Schedule, 2nd Edition, and Autism Diagnostic Interview-Revised).

	MSDD Group (n = 36)	ASD Group (n = 108)	*p*-Value
**ADOS-2, mean (SD)**			
CSS	3.94 (1.45)	6.82 (1.86)	<0.01 *
**ADI-R, mean (SD)**			
Reciprocal social interaction	6.05 (3.62)	14.70 (6.19)	<0.01 *
Communication and language	6.25 (3.57)	11.09 (3.14)	<0.01 *
Restricted, repetitive, and stereotyped behavior	1.85 (1.53)	4.43 (2.49)	<0.01 *

MSDD = mixed specific developmental disorder; ASD = autism spectrum disorder; SD = standard deviation; ADOS-2 = Autism Diagnostic Observation Schedule, 2nd Edition; CSS = calibrated severity score; ADI-R = Autism Diagnostic Interview-Revised. * *p* < 0.05.

## Data Availability

The original contributions presented in the study are included in the article; further inquiries can be directed to the corresponding authors. Additional data are not publicly available due to privacy concerns.

## Supplementary Materials

The following supporting information can be downloaded at https://www.mdpi.com/article/10.3390/children12020125/s1: Figure S1: Developmental patterns in clinical groups.
