# Supplementary material for: Developmental Patterns in Autism and Other Neurodevelopmental Disorders in Preschool Children
Source: Children (Basel). 2025 Jan 24;12(2):125. doi: 10.3390/children12020125 (PMC11854899; doi:10.3390/children12020125)
Supplement: Supplementary file 1 [file children-12-00125-s001.zip › children-3429408-supplementary.pdf]

Figure S1. Developmental patterns in clinical groups.

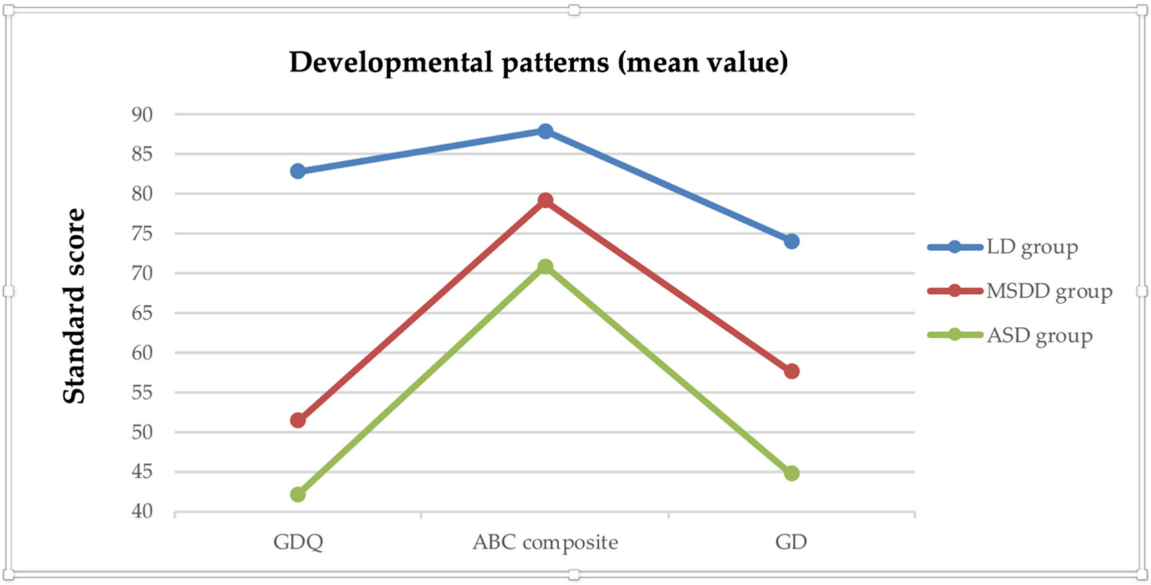

LD = language disorder; MSDD = mixed specific developmental disorder; ASD = autism spectrum disorder; GDQ = general developmental quotient (Griffiths-III); ABC = adaptive behaviour composite (VABS-II); GD = general development (DP-3).
